# PD-1 Immune Checkpoint Blockade and PSGL-1 Inhibition Synergize to Reinvigorate Exhausted T Cells

**DOI:** 10.3389/fimmu.2022.869768

**Published:** 2022-06-14

**Authors:** Karla M. Viramontes, Emily N. Neubert, Julia M. DeRogatis, Roberto Tinoco

**Affiliations:** ^1^ Department of Molecular Biology and Biochemistry, School of Biological Sciences, University of California, Irvine, Irvine, CA, United States; ^2^ Center for Virus Research, University of California, Irvine, Irvine, CA, United States

**Keywords:** PD-1, PSGL- 1, immune checkpoint inhibitors, LCMV (lymphocytic choriomeningitis virus), melanoma, T cell exhaustion, immune checkpoints, chronic infections

## Abstract

Chronic viral infections where the antigen persists long-term, induces an exhaustion phenotype in responding T cells. It is now evident that immune checkpoints on T cells including PD-1, CTLA-4, and PSGL-1 (*Selplg*) are linked with the differentiation of exhausted cells. Chronic T cell receptor signaling induces transcriptional signatures that result in the development of various exhausted T cell subsets, including the stem-like T cell precursor exhausted (Tpex) cells, which can be reinvigorated by immune checkpoint inhibitors (ICIs). While PSGL-1 has been shown to inhibit T cell responses in various disease models, the cell-intrinsic function of PSGL-1 in the differentiation, maintenance, and reinvigoration of exhausted T cells is unknown. We found *Selplg^-/-^* T cells had increased expansion in melanoma tumors and in early stages of chronic viral infection. Despite their increase, both WT and *Selplg^-/-^* T cells eventually became phenotypically and functionally exhausted. Even though virus-specific *Selplg^-/-^* CD4^+^ and CD8^+^ T cells were increased at the peak of T cell expansion, they decreased to lower levels than WT T cells at later stages of chronic infection. We found that *Selplg^-/-^* CD8^+^ Tpex (SLAMF6^hi^TIM3^lo^, PD-1^+^TIM3^+^, TOX^+^, TCF-1^+^) cell frequencies and numbers were decreased compared to WT T cells. Importantly, even though virus-specific *Selplg^-/-^* CD4^+^ and CD8^+^ T cells were lower, they were reinvigorated more effectively than WT T cells after anti-PD-L1 treatment. We found increased *SELPLG* expression in Hepatitis C-specific CD8^+^ T cells in patients with chronic infection, whereas these levels were decreased in patients that resolved the infection. Together, our findings showed multiple PSGL-1 regulatory functions in exhausted T cells. We found that PSGL-1 is a cell-intrinsic inhibitor that limits T cells in tumors and in persistently infected hosts. Additionally, while PSGL-1 is linked with T cell exhaustion, its expression was required for their long-term maintenance and optimal differentiation into Tpex cells. Finally, PSGL-1 restrained the reinvigoration potential of exhausted CD4^+^ and CD8^+^ T cells during ICI therapy. Our findings highlight that targeting PSGL-1 may have therapeutic potential alone or in combination with other ICIs to reinvigorate exhausted T cells in patients with chronic infections or cancer.

## Introduction

Viral pathogens have evolved multiple mechanisms to evade the immune response and prevent their elimination by the host. While T lymphocytes are key players that mediate destruction of virally infected cells during chronic viral infections such as in Hepatitis B and C, human immunodeficiency virus (HIV), and chronic *Lymphocytic choriomeningitis* (LCMV) viral infection, the pathogen is never eliminated (1). The persisting antigen induces a state of T cell exhaustion characterized by diminished effector cytotoxic and helper functions (1). T cell exhaustion is a complex process that is still not fully understood. However, the LCMV clone 13 (Cl13) chronic infection model has helped uncover some of the phenotypic and functional changes and mechanisms that drive T cell exhaustion (2–5). Exhausted T cells are characterized by their upregulated and sustained expression of immune inhibitory receptors such as PD-1, CTLA-4, TIM-3, LAG-3, PSGL-1 and many others (6–8). Importantly, the upregulation of these inhibitory receptors promotes T cell dysfunction by diminishing T cell receptor (TCR) signaling (9). Subsets of exhausted CD8^+^ T cells have also been identified including stem-like progenitor exhausted T cells (Tpex) and terminally exhausted T cells (Tex) (10, 11). Tpex cells can self-renew and give rise to Tex cells, and although less functional than effector T cells arising from acute viral infection, Texs retain sufficient function to restrain viral replication (12–14). Importantly, in the context of immune checkpoint inhibitors (ICIs), Tpex cells are the cells that respond to these treatments as shown by their increased proliferation and expansion (11). Furthermore, Tpex expansion after ICI therapy subsequently results in the increase in Tex cells, which have higher cytotoxic functions that mediate antigen clearance (11).

In addition to CD8^+^ T cells, CD4^+^ T cells are also functionally exhausted during chronic viral infection and their helper functions are compromised (15). Both CD4^+^ and CD8^+^ T cells upregulate and express high levels of inhibitory receptors and lose their effector functions (16, 17). This impaired effector function is evident in the sequential loss of anti-viral cytokine production, including IFN-γ, TNF-α, and IL-2 (18, 19). Limited IL-2 production by CD4^+^ T cells is detrimental to the adaptive immune response as this cytokine is critical for sustaining CD8^+^ T cells during viral infection (20, 21). While CD4^+^ T cells initially differentiate towards a Th1 phenotype during Cl13 infection, they eventually transition and acquire a Tfh phenotype at late stages of chronic viral infection (22). Virus-specific CD4^+^ Tfh cells produce IL-21 which sustains CD8^+^ T cell responses and stimulates antibody production, which together decrease viremia (22, 23). Although exhausted, CD4^+^ T cells continue to assist in the anti-viral response, as evidenced by the life-long viremia observed in Cl13 infected mice that are depleted of CD4^+^ T cells (15).

While the mechanisms of T cell exhaustion are not fully known, the ability to reinvigorate these cells is of great clinical interest. Indeed, T cell function can be improved to promote resolution of chronic viral infections (19, 24, 25). Moreover, immune checkpoint inhibitors (ICIs) targeting CTLA-4 and PD-1/PD-L1 to reinvigorate exhausted T cells can improve immunity against tumors (26, 27). ICIs have had impressive clinical success and are now standard therapies for multiple cancer types including metastatic melanoma (28). While ICI therapy is promising, few cancer patients respond to current treatments and ICI resistance mechanisms are currently being investigated (29). Recent studies in this area have found molecular mechanism of “scarring” in exhausted T cells, in which permanent transcriptional and epigenetic changes sustain their dysfunctional phenotypes (30–32). The differentiation and maintenance of the T cell exhausted state and the molecular players regulating this process continue to be an area of clinical interest since ICIs aim to reverse this phenotype.

P-selectin glycoprotein ligand-1 (PSGL-1, *Selplg*) is a transmembrane protein that is expressed on all hemopoietic cells, including myeloid and lymphoid cells (33). Although PSGL-1 was initially known for its role in cellular migration through engaging selectins (34), PSGL-1 has been identified as a new immune checkpoint which can inhibit T cells (8, 35). Furthermore, PSGL-1 on T cells was recently shown to engage V-domain Ig suppressor of T cell activation (VISTA) which can inhibit T cell proliferation and promote tumor progression (36, 37). PSGL-1 inhibitory functions were observed in *Selplg^-/-^* Cl13-infected mice, where virus-specific CD4^+^ and CD8^+^ T cells were increased, had decreased inhibitory receptor expression, and increased effector functions which led to viral clearance (8). *Selplg^-/-^* mice were also shown to have increased anti-tumor immunity to melanoma (8). Additionally, during acute viral infection, *Selplg^-/-^* T cells were increased at both the effector and memory stage (38). However, the cell-intrinsic PSGL-1 function in the differentiation of exhausted CD4^+^ and CD8^+^ T cells and its role during the reinvigoration process after ICI therapy is unknown.

In this study, we found a cell-intrinsic role for PSGL-1 expression in the maintenance of exhausted CD4^+^ and CD8^+^ T cells. We observed a PSGL-1 inhibitory function in CD8^+^ T cells as shown by the increased expansion of *Selplg^-/-^* CD8^+^ T cells in B16-GP_33_ melanoma tumors. During Cl13 infection, *Selplg^-/-^* CD4^+^ and CD8^+^ T cells increased at early stages of Cl13 infection, however, these cells eventually decreased to lower levels than those of WT T cells at later stages of chronic infection. We found that *Selplg^-/-^* CD8^+^ T cells had a decrease in the frequencies of Tpex stem-like cells. While both WT and *Selplg^-/-^* CD4^+^ and CD8^+^ T cells eventually became functionally exhausted, *Selplg^-/-^* T cells had superior reinvigoration after anti-PD-L1 treatment. Finally, we found that PSGL-1 gene expression was increased in virus-specific CD8^+^ T cells from chronically infected Hepatitis C patients and decreased in virus-specific CD8^+^ T cells from Hepatitis C spontaneous resolvers. Our findings highlight an important function of the PSGL-1 immune checkpoint in regulating the expansion, maintenance, and reinvigoration of exhausted T cells.

## Results

### Virus-Specific *Selplg* ^-/-^ CD4^+^ and CD8^+^ T Cells Initially Expand but Decrease Over the Course of Chronic Viral Infection

**Figure 1:**
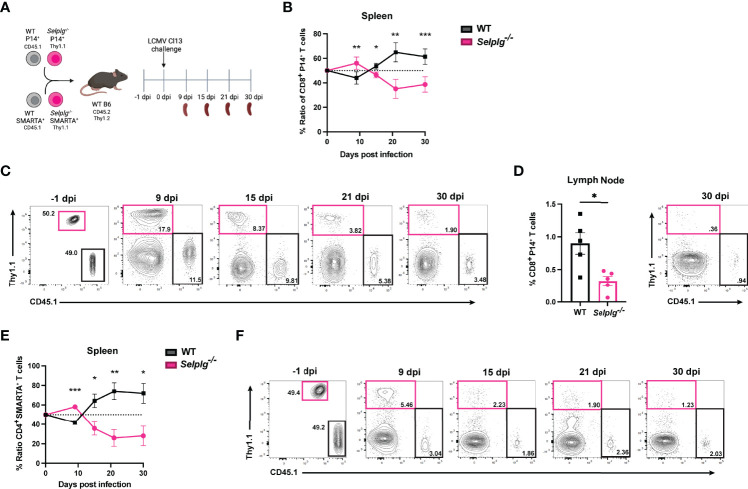
*Selplg^-/-^* CD4^+^ and CD8^+^ T cell kinetics over the course of chronic viral infection. WT and *Selplg* ^-/-^ P14 and SMARTA T cells were adoptively transferred at equal ratios into naïve WT recipients and infected with Cl13 one day after **(A)**. Ratio of WT and *Selplg* ^-/-^ P14 T cells in the spleen at the indicated timepoints **(B)** and representative FACs plots **(C)**. Frequencies of WT and *Selplg* ^-/-^ P14 T cells in the lymph nodes at 30dpi **(D)**. Ratio of WT and *Selplg* ^-/-^ SMARTA T cells in the spleen **(E)** and representative FACs plots **(F)**. *p < 0.05, **p < 0.01, ***p < 0.001, (unpaired *t*-test). Data are representative of four independent experiments all with five or more mice per group (error bars, s.e.m.).

To investigate the cell-intrinsic role of PSGL-1 in T cells over the course of chronic viral infection, we co-injected small numbers (1-2 x10^3^) of TCR transgenic (Tg) CD4^+^ and CD8^+^ T cells specific for LCMV into WT mice. Naïve WT (CD45.1^+^) and *Selplg* ^-/-^ (Thy1.1^+^) P14^+^ CD8^+^ and WT (CD45.1^+^) and *Selplg* ^-/-^ (Thy1.1^+^) SMARTA^+^ CD4^+^ T cells were adoptively transferred in WT (CD45.2^+^Thy1.2^+^) hosts at 1:1 equal ratio (**Figure 1A**). After one day, mice were infected with LCMV Cl13 and T cell responses were analyzed at 9, 15, 21, and 30 days post-infection (dpi) **(** **Figure 1A** **)**. We compared the frequencies of WT and *Selplg* ^-/-^ P14^+^ CD8^+^ T cells in the spleen and observed a higher frequency and ratio of *Selplg^-/-^* P14^+^ T cells to WT at 9dpi, however, *Selplg^-/-^* P14^+^ T cell frequencies decreased by 15dpi and remained lower than WT at 30dpi **(** **Figures 1B, C** **)**. We also observed lower frequencies of *Selplg* ^-/-^ P14^+^ T cells compared to WT T cells in lymph nodes at 30dpi **(** **Figure 1D** **)**. We next examined the ratio and frequencies of CD4^+^ T cells and observed an initial increase in *Selplg* ^-/-^ SMARTA^+^ CD4^+^ T cells at 9dpi, which then significantly decreased throughout infection compared to WT T cells (**Figures 1E, F**). These findings showed the cell-intrinsic PSGL-1 inhibitory function in virus-specific T cells at the peak of anti-viral T cell expansion and revealed that virus-specific CD4^+^ and CD8^+^ T cells required PSGL-1 expression for their long-term maintenance during chronic viral infection.

### Effector Function of *Selplg^-/-^* T Cells at Early and Late Stages of Chronic Viral Infection

**Figure 2:**
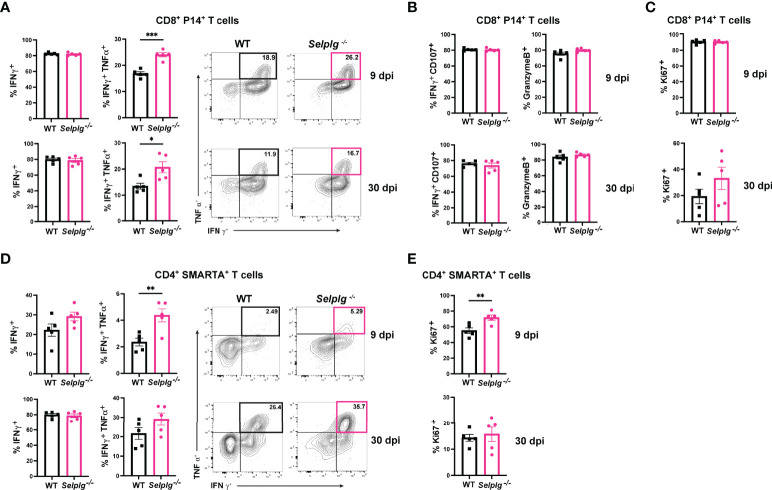
PSGL-1-deficient CD4^+^ and CD8^+^ T cell effector functions. Spleens were isolated from Cl13 infected mice at 9 and 30dpi and *ex vivo* stimulated with cognate peptide. Frequency of IFN-γ^+^ and IFN-γ^+^ TNF-α^+^ of WT and *Selplg* ^-/-^ P14^+^ T cells **(A)**. Frequencies of IFN-γ^+^ CD107^+^ and GranzymeB^+^ **(B)** and Ki67^+^ **(C)** WT and *Selplg* ^-/-^ P14^+^ T cells. Frequencies of IFN-γ^+^ and IFN-γ^+^TNF-α^+^ WT and *Selplg* ^-/-^ SMARTA^+^ T cells **(D)**. Frequencies of Ki67^+^ WT and *Selplg* ^-/-^ SMARTA^+^ T cells **(E)**. *p < 0.05, **p < 0.01, ***p < 0.001, (unpaired *t*-test). Data are representative of four independent experiments all with five or more mice per group (error bars, s.e.m.).

**Figure 3:**
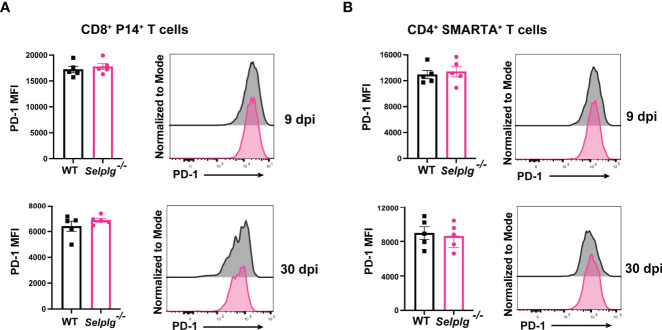
*Selplg* ^-/-^ CD4^+^ and CD8^+^ T cells express high PD-1 levels. Spleens were isolated from Cl13 infected mice at day 9 and 30dpi. Mean fluorescence intensity (MFI) and representative histogram of PD-1 expression levels on WT and *Selplg* ^-/-^ P14^+^ T cells **(A)** and SMARTA^+^ T cells **(B)**. Data are representative of four independent experiments all with five or more mice per group (error bars, s.e.m.).

We next characterized the exhaustion phenotype in T cells during chronic viral infection. We first assessed *ex vivo* cytokine production in the spleen of infected mice and observed similar frequencies of IFN-γ^+^ WT and *Selplg^-/-^* P14^+^ T cells at 9 and 30dpi (**Figure 2A**). Exhausted T cells are characterized by their loss of polyfunctionality, and we confirmed a decrease in IFN-γ^+^TNF-α^+^ production by both WT and *Selplg^-/-^* T cells, however *Selplg^-/-^* P14^+^ T cells had a slight increase in these cytokine^+^ cells at 9 and 30dpi (**Figure 2A**). We further characterized CD8^+^ T cell effector functions and observed high expression of IFN-γ^+^CD107^+^ and GranzymeB^+^ T cells, with no differences between WT or *Selplg^-/-^* P14^+^ T cells at 9 or 30dpi (**Figure 2B**). We evaluated proliferation by Ki67 staining and observed up to ~90% Ki67^+^ P14^+^ T cells at 9dpi and ~20% Ki67^+^ P14^+^ T cells at 30dpi, noting no differences between WT and *Selplg^-/-^* P14^+^ T cells (**Figure 2C**). We next evaluated functional changes in CD4^+^ T cells and observed similar IFN-γ production in both WT and *Selplg^-/-^* SMARTA^+^ T cells at 9dpi (**Figure 2D**). Although IFN-γ^+^ cells increased by 30dpi, there were no differences between WT and *Selplg^-/-^* T cells (**Figure 2D**). We observed a slight increase in IFN-γ^+^TNF-α^+^ *Selplg^-/-^* SMARTA^+^ T cells at 9dpi, however these levels were like WT T cells at 30dpi (**Figure 2D**). *Selplg^-/-^* SMARTA^+^ T cells had increased Ki67^+^ cells at 9dpi, but no differences were observed between WT and *Selplg^-/-^* SMARTA^+^ T cells at 30dpi (**Figure 2E**). We next evaluated PD-1 expression on both P14^+^ and SMARTA^+^ T cells and observed high PD-1 expression at 9dpi, with no differences between WT and *Selplg^-/-^* T cells (**Figures 3A, B**). Although PD-1 expression decreased by 30dpi compared to 9dpi, expression levels remained high, with no differences between WT and *Selplg^-/-^* T cells (**Figures 3A, B**). These findings showed that P14^+^ and SMARTA^+^ *Selplg^-/-^* T cells are phenotypically and functionally exhausted to similar levels as WT T cells by 30dpi.

### *Selplg* ^-/-^ Tpex CD8^+^ T Cells Are Decreased During Chronic Viral Infection

**Figure 4:**
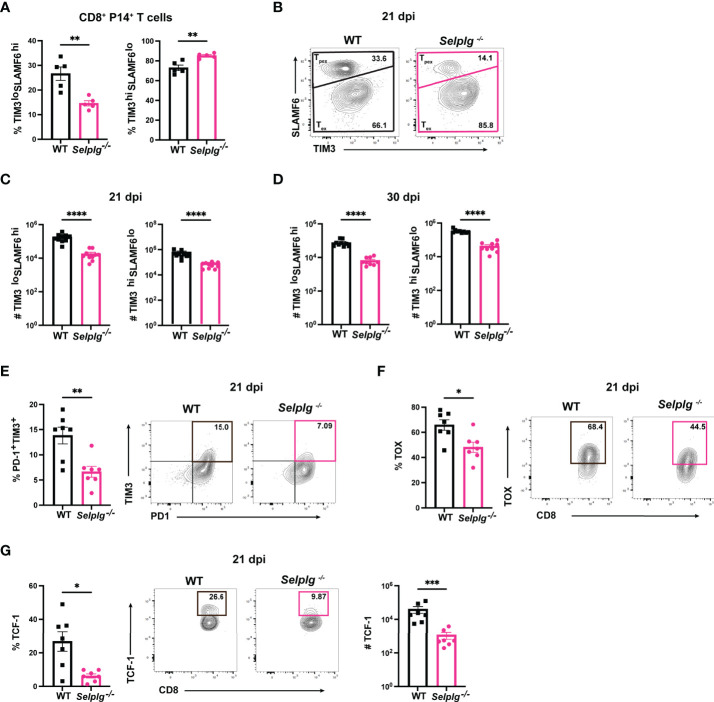
PSGL-1 deficient progenitor exhausted CD8^+^ T cells (Tpex) are decreased during chronic viral infection. Spleens were isolated from Cl13 infected mice at 21 and 30dpi. Frequencies of Tpex and Tex in WT and *Selplg* ^-/-^ P14^+^ T cells at 21 dpi **(A)** and representative FACs plots **(B)**. Numbers of Tpex and Tex of WT and *Selplg* ^-/-^ P14 T cells at 21dpi **(C)** and 30dpi **(D)**. Frequencies of PD-1^+^TIM3^+^ **(E)**, TOX^+^ **(F)**, and TCF-1^+^ **(G)** T cells at 21dpi. **p < 0.01, ****p < 0.001, (unpaired *t*-test). Data are representative of four independent experiments all with five or more mice per group (error bars, s.e.m.). * < 0.05, *** < 0.005.

Since we observed a decrease in the frequencies of *Selplg* ^-/-^ P14^+^ cells by 30dpi (**Figure 1**), we next determined whether this decrease was due to changes in Tpex or Tex cells. Using the same co-transfer approach, we analyzed the frequencies of Tpex (SLAMF6^hi^TIM3^lo^) and Tex (SLAMF6^lo^TIM3^hi^) cells in WT and *Selplg* ^-/-^ P14^+^ T cells. We observed a decrease in Tpex frequencies and a slight increase in Tex frequencies in *Selplg^-/-^* P14^+^ T cells at 21dpi (**Figures 4A, B**). We quantified the absolute number of Tpex and Tex cells in the spleens of infected mice and found decreased numbers of Tpex and Tex in *Selplg^-/-^* P14^+^ T cells compared to WT T cells at 21 and 30dpi (**Figures 4C, D**). We observed decreased frequencies of PD-1^+^TIM3^+^, TOX^+^, and TCF-1^+^ *Selplg^-/-^* P14^+^ T cells at 21dpi, which are also markers of Tpex cells (**Figures 4E–G**). These findings showed that in *Selplg^-/-^* P14^+^ T cells, frequencies of Tpex cells were decreased, which may result in the decreased maintenance of the exhausted *Selplg^-/-^* T cells over the course of chronic infection.

### PSGL-1 Deletion in CD8^+^ T Cells Combined With Anti-PD-L1 Treatment Can Synergize to Reinvigorate Exhausted T Cells

**Figure 5:**
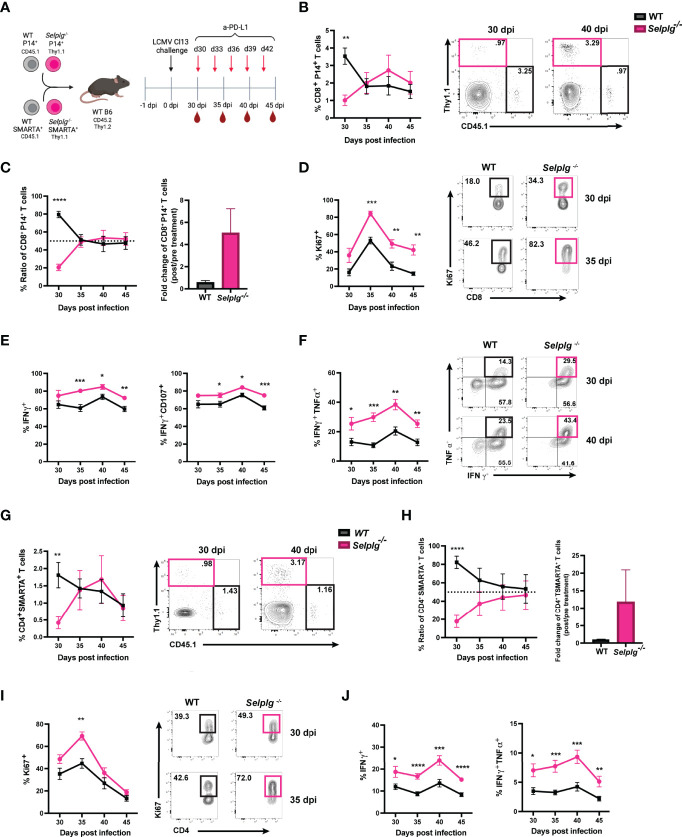
PD-L1 blockade synergizes to reinvigorate *Selplg* ^-/-^ CD4^+^ and CD8^+^ T cells. Anti-PD-L1 blockade experimental approach **(A)**. Frequencies of WT and *Selplg* ^-/-^ P14^+^ T cells before (30dpi) and after anti-PD-L1 treatment **(B)**. Ratio and fold change of WT and *Selplg* ^-/-^ P14^+^ T cells before (30dpi) and after (40dpi) anti-PD-L1 treatment **(C)**. Frequencies of Ki67^+^ **(D)**, IFN-γ^+^ and IFN-γ^+^ CD107^+^ **(E)**, and IFN-γ^+^ TNF-α^+^ **(F)** of WT and *Selplg* ^-/-^ P14^+^ T cells. Frequencies of WT and *Selplg* ^-/-^ SMARTA^+^ T cells before (30dpi) and after anti-PD-L1 treatment **(G)**. Ratio and fold change of WT and *Selplg* ^-/-^ SMARTA T cells before (30dpi) and after (40dpi) anti-PD-L1 treatment **(H)**. Frequencies of Ki67^+^ **(I)** and IFN-γ^+^ and IFN-γ^+^ TNF-α^+^ **(J)** of WT and *Selplg* ^-/-^ SMARTA^+^ T cells. *p < 0.05, **p < 0.01, ***p < 0.005 ****p < 0.001, (unpaired *t*-test). Data are representative of three independent experiments all with five or more mice per group (error bars, s.e.m.).

We next evaluated whether blocking the PD-1 pathway reinvigorated the response of exhausted *Selplg^-/-^* P14^+^ T cells. We co-transferred WT and *Selplg^-/-^* P14^+^ T cells in WT mice, infected with Cl13, and injected either IgG or anti-PD-L1 starting at 30dpi and every 3 days to 42dpi (**Figure 5A**). We evaluated T cell frequencies and phenotype in blood before (30dpi) and during antibody treatment (**Figure 5A**). We observed a significantly higher frequency of WT P14^+^ T cells (~3.5%) than *Selplg^-/-^* P14^+^ cells (~1%) at 30dpi before antibody treatment (**Figure 5B**). However, at 35dpi, the frequency of *Selplg^-/-^* P14^+^ T cells increased to similar levels as WT P14^+^ cells and remained higher to 45dpi (**Figure 5B**). Since WT and *Selplg^-/-^* P14^+^ T cells were initially injected at a 1:1 ratio, we determined ratio and fold change throughout the course of anti-PD-L1 treatment. At 30dpi, WT P14^+^ T cells represented ~80% of the transferred population while *Selplg^-/-^* P14^+^ T cells were ~20% (**Figure 5C**). However, by 35dpi and onwards, *Selplg^-/-^* P14^+^ T cells increased to ~50% of the transferred cells, and they remained increased at 45dpi (**Figure 5C**). We determined the fold change of P14^+^ T cells before and after treatment and observed that *Selplg^-/-^* P14^+^ T cells had a greater fold change from pre-treatment compared to WT P14^+^ T cells (**Figure 5C**). Since we observed an increase in *Selplg^-/-^* P14^+^ T cells after anti-PD-L1 treatment, we determined whether there was a difference in their proliferation compared to WT T cells. Although *Selplg^-/-^* P14^+^ trended for higher Ki67^+^ cells, there was no statistical significance compared to WT T cells pre-treatment (30dpi) (**Figure 5D**). However, after anti-PD-L1 treatment, Ki67^+^ WT P14^+^ T cells increased from ~18% pre-treatment to ~46% by 35dpi and decreased thereafter (**Figure 5D**). Ki67^+^ *Selplg^-/-^* P14^+^ T cells increased from ~34% pre-treatment to ~82% by 35dpi and were significantly higher than WT T cells at 45dpi (**Figure 5D**). We also observed a significant increase in the frequencies of IFN-γ^+^, IFN-γ^+^CD107^+^, and IFN-γ^+^TNF-α^+^ *Selplg^-/-^* P14^+^ T cells during anti-PD-L1 treatment (**Figures 5E, F**). These findings showed that anti-PD-L1 treatment reinvigorated the proliferation and effector function of exhausted *Selplg^-/-^* P14^+^ T cells more than WT P14^+^ T cells.

### *Selplg* ^-/-^ CD4^+^ T Cells Were Reinvigorated After Anti-PD-L1 Treatment

We next determined whether anti-PD-L1 treatment changed *Selplg^-/-^* SMARTA^+^ T cell responses. WT and *Selplg^-/-^* SMARTA^+^ T cells were co-transferred into WT mice, which were then infected with Cl13 (**Figure 5A**). T cells were analyzed in the blood at 30dpi, before IgG and anti-PD-L1 injections, and during antibody treatment (**Figure 5A**). We observed significantly lower frequencies of *Selplg^-/-^* SMARTA^+^ T cells than WT T cells at 30dpi, before anti-PD-L1 treatment (**Figure 5G**). However, after anti-PD-L1 treatment, *Selplg^-/-^* SMARTA^+^ T cells increased to similar frequencies as WT T cells (**Figure 5G**). WT and *Selplg^-/-^* SMARTA^+^ T cells were co-transferred at a 1:1 ratio, but this ratio changed to 4:1 by 30dpi (**Figure 5H**). *Selplg^-/-^* SMARTA^+^ T cells increased after anti-PD-L1 treatment and approached a 1:1 ratio with WT T cells at 45dpi (**Figure 5H**). In addition, *Selplg^-/-^* SMARTA^+^ T cells had a higher fold change from pre-treatment than WT SMARTA^+^ T cells (**Figure 5H**). We next assessed proliferation and observed a significant increase in Ki67^+^ *Selplg^-/-^* SMARTA^+^ T cells at 35dpi with no differences at later timepoints (**Figure 5I**). We observed a significant increase in IFN-γ^+^ and IFN-γ^+^TNF-α^+^ in *Selplg^-/-^* SMARTA^+^ T cells which peaked at 40dpi (**Figure 5J**). These findings showed that anti-PD-L1 treatment increased the accumulation, proliferation, and effector function of exhausted *Selplg^-/-^* SMARTA^+^ T cells more than WT T cells.

### *Selplg^-/-^* CD8^+^ T Cells Are Increased in Melanoma Tumors

**Figure 6:**
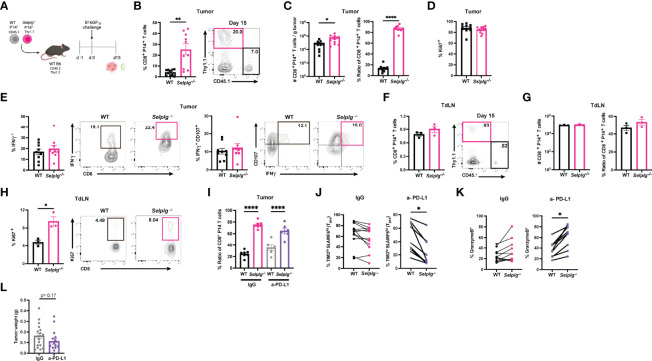
PSGL-1-deficient CD8^+^ T cells are increased in melanoma tumors. WT and *Selplg* ^-/-^ P14 T cells were adoptively transferred at equal ratios into naïve WT recipients and injected with B16-GP_33_ tumors s.c. one day later **(A)**. Frequencies of WT and *Selplg* ^-/-^ P14^+^ T cells in tumor **(B)**. T cell numbers per gram of tumor and ratio of WT and *Selplg* ^-/-^ P14^+^ T cells **(C)**. Frequencies of Ki67^+^ **(D)**, IFN-γ^+^ and IFN-γ^+^ CD107^+^ **(E)** of WT and *Selplg* ^-/-^ P14^+^ T cells. Frequencies of WT and *Selplg* ^-/-^ P14^+^ T cells in pooled tumor draining lymph nodes (TdLN) **(F)**. Numbers and ratio **(G)** and Ki67^+^ **(H)** of WT and *Selplg* ^-/-^ P14^+^ T cells in pooled TdLNs. Ratio of T cells **(I)**, Tpex frequencies **(J)**, and GranzymeB^+^ T cells **(K)**, and tumor mass **(L)** in IgG or anti-PD-L1 treated mice. *p < 0.05, **p < 0.01, ****p < 0.001, (unpaired *t*-test). Data are representative of three independent experiments all with nine or more mice per group (error bars, s.e.m.).

We next assessed whether PSGL-1 expression impacted the CD8^+^ T cell response in melanoma tumors. We co-transferred WT and *Selplg^-/-^* P14^+^ T cells at a 1:1 ratio in WT mice and one day later, subcutaneously injected B16-GP_33_ melanoma cells (**Figure 6A**). We observed increased frequencies of *Selplg* ^-/-^ P14^+^ T cells in tumors compared to WT T cells 15 days after tumor injection (**Figure 6B**). Consistent with the increased frequencies, we also observed increased numbers of *Selplg^-/-^* P14^+^ cells per gram of tumor (**Figure 6C**). We noted that the 1:1 pre-injection T cell ratio changed to 1:4 in tumors, with the increase of *Selplg^-/-^* P14^+^ T cells (**Figure 6C**). We observed no differences in proliferation of WT or *Selplg^-/-^* P14^+^ T cells in tumors as measured by Ki67^+^ cells (**Figure 6D**). We also noted that both WT and *Selplg^-/-^* P14^+^ T cells in tumors had similar frequencies of IFN-γ^+^ and IFN-γ^+^CD107a^+^ T cells (**Figure 6E**). We next evaluated donor T cell frequencies in pooled tumor draining lymph nodes (TdLN) and observed similar frequencies of WT and *Selplg* ^-/-^ P14^+^ T cells (**Figure 6F**). Furthermore, there were no differences in cell numbers or the ratio of WT to *Selplg^-/-^* P14^+^ T cells, in TdLN (**Figure 6G**). We did note a significant increase in Ki67^+^ cells in *Selplg^-/-^* P14^+^ T cells in the TdLN (**Figure 6H**). We next evaluated whether anti-PD-L1 treatment changed the frequencies of WT and *Selplg^-/-^* P14^+^ T cells and found similar ratios between IgG and anti-PD-L1 treated mice (**Figure 6I**). We observed that compared to IgG treated mice, anti-PD-L1 treatment decreased the frequencies of *Selplg^-/-^* SLAMF6^hi^TIM3^lo^ (Tpex) cells (**Figure 6J**). We observed increased GranzymeB^+^ *Selplg^-/-^* P14^+^ T cells after anti-PD-L1 treatment (**Figure 6K**), and no significant differences in tumor mass between IgG and anti-PD-L1 treated mice (**Figure 6L**). These findings showed that *Selplg^-/-^* P14^+^ T cells were increased in B16-GP_33_ melanoma tumors.

### *SELPLG* Is Increased in HCV-Specific CD8^+^ T Cells During Chronic Hepatitis C Viral Infection

**Figure 7:**
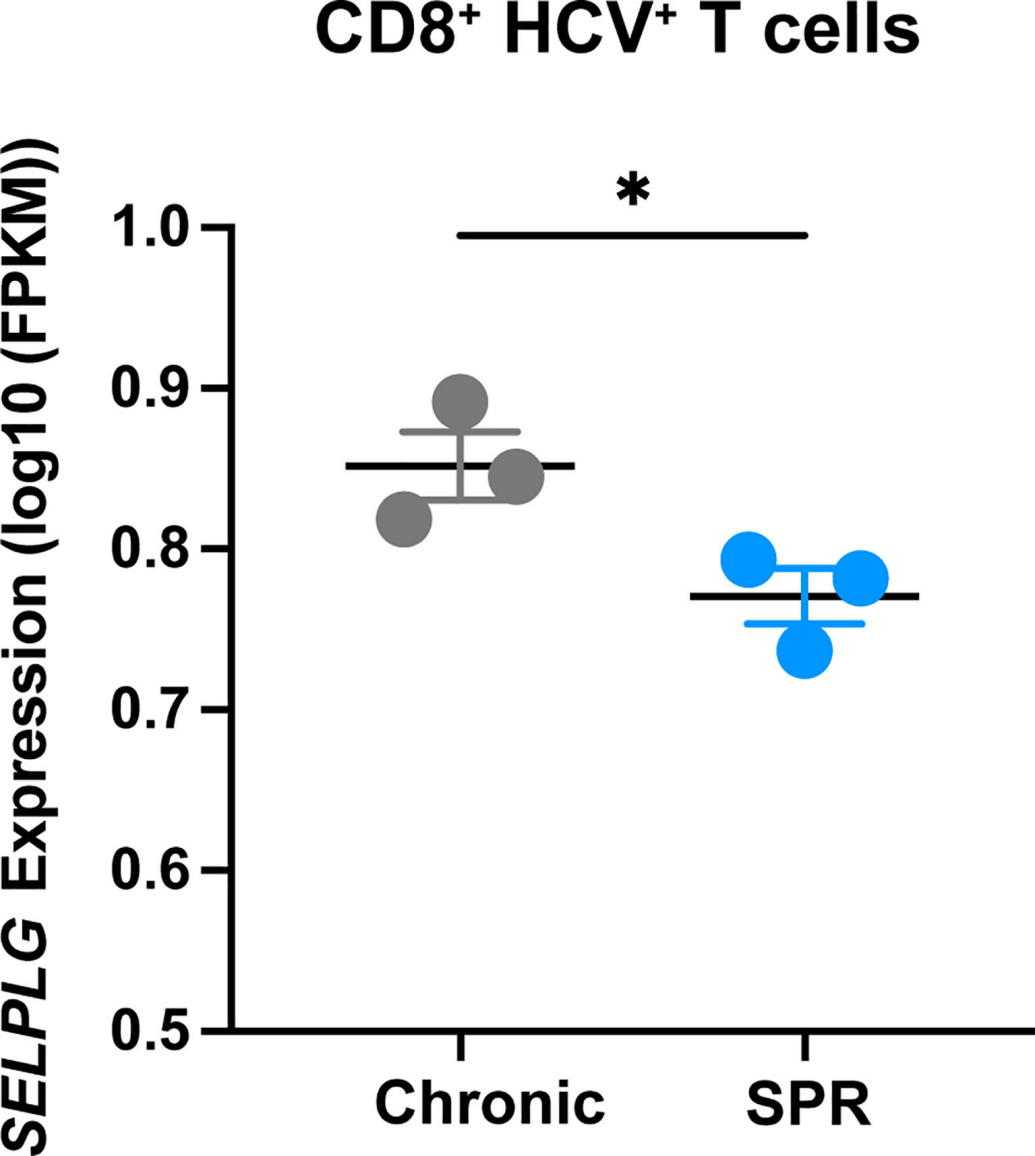
*SELPLG* is upregulated in CD8^+^ T cells in patients with chronic HCV. Fragments per kilo base per million mapped reads (FPKMs) of *SELPLG* expression in HCV-specific CD8^+^ T cells isolated from blood of chronically HCV infected patients or patients who spontaneously resolved their infection (SPR). *p < 0.05 (unpaired *t*-test). Each dot represents one patient (error bars, s.e.m.).

We next determined the relevance of PSGL-1 (*SELPLG*) expression in CD8^+^ T cells from patients with Hepatitis C infection and analyzed RNA sequencing data from the Hensel et al. study (31). In short, low-input RNA sequencing was performed on HCV-specific CD8^+^ T cells from blood of patients infected with chronic Hepatitis C virus (cHCV) and HCV patients who were spontaneous resolvers (SPR) that cleared the viral infection. We observed significantly increased *SELPLG* expression in CD8^+^ T cells from cHCV patients compared to SPR patients (**Figure 7**). These findings showed that *SELPLG* was highly expressed in CD8^+^ T cells during chronic Hepatitis C viral infection and was decreased when the virus was eliminated from the host.

## Discussion

While T cell immune checkpoints are known to prevent antigen clearance during chronic viral infection and cancer, they have important inhibitory functions that fine-tune T cell receptor signals and thresholds that shape T cell differentiation (39). Studies showed that PD-1 and CTLA-4 expression were important in T cell differentiation since deficiency in these immune checkpoints resulted in their acquisition of aberrant cellular states (39). Furthermore, a cell-intrinsic role for PD-1 expression in P14^+^ CD8^+^ T cells was shown to be necessary for their long-term maintenance during Cl13 infection (40). Like this PD-1 study, we also found that cell-intrinsic PSGL-1 expression was required for the long-term maintenance of P14^+^ T cells during Cl13 infection, as *Selplg^-/-^* P14^+^ T cells were decreased at late stages of viral infection. Furthermore, *Selplg^-/-^* P14^+^ T cells showed defective differentiation as these cells had decreased frequencies of Tpex cells which sustain the exhausted T cell population long-term. Tpex cells have been shown to be stem-like and survive to give rise to Tex cells, which although destined to die, retain some effector functions that restrain viral replication (41). Even though these exhausted T cells are dysfunctional, they continue to provide limited protection to the chronically infected host (42). We found that numerically, *Selplg^-/-^* P14^+^ Tpex cells were decreased at late stages of Cl13 infection, indicating that this defective differentiation correlated with their decrease over the course of infection (41). Since *Selplg^-/-^* P14^+^ T cells drastically declined during Cl13 infection compared to WT T cells and we observed no differences between their proliferation suggests that *Selplg^-/-^* P14^+^ T cells had a decreased ability to survive, which could also be explained by their diminished Tpex population. This contrasts with what was observed in *Selplg^-/-^* mice infected with Arm or Cl13 where T cell survival was increased, in part due to the viral clearance that occurs in *Selplg^-/-^* infected mice (8, 38). The importance of immune checkpoints in T cell differentiation was also highlighted in studies using WT and *Selplg^-/-^* SMARTA^+^ and P14^+^ T cells transferred in WT Arm infected mice, which showed that even though more memory *Selplg^-/-^* TCR transgenic T cells developed, they failed to be recalled during a secondary infection (38). A similar phenotype in defective memory differentiation was also observed in PD-1-deficient T cells responding to respiratory viral infection (43). While these prior findings were observed during acute viral infection, our findings now revealed that cell-intrinsic PSGL-1 expression also regulated exhausted CD8^+^ T cell differentiation during chronic viral infection. Our approach examining the differentiation of these transferred *Selplg^-/-^* T cells in a host that is chronically infected with persistent antigen showed an important function for PSGL-1 expression in the differentiation and maintenance of these cells.

ICI efficacy to combat cancers and chronic viral infections is achieved through their ability to reinvigorate exhausted T cells (26, 27, 44–46). The restoration of effector functions in exhausted T cells can lead to control of chronic viral infection as observed after anti-PD-1 and anti-CTLA-4 treatment (46). Furthermore, targeting PD-1 and CTLA-4 in the clinic has shown efficacy in cancer patients, with these now being the standard of care in melanoma (47, 48). While current ICIs have shown some efficacy, most patients continue to be unresponsive due to multiple resistance mechanisms (49–51). Here we show that the combination of anti-PD-L1 and PSGL-1-deficiency synergized to reinvigorate exhausted virus-specific CD4^+^ and CD8^+^ T cells, as shown by their greater expansion and function than WT T cells. The Tpex subset is the key cell-type responding to anti-PD-1/PD-L1 blockade (41). For *Selplg^-/-^* CD8^+^ T cells, it is remarkable that prior to anti-PD-L1 treatment, *Selplg^-/-^* Tpex cells were significantly decreased compared to WT Tpex cells. We observed that despite this disadvantage, *Selplg^-/-^* CD8^+^ T cells had a more significant expansion than WT T cells during anti-PD-L1 therapy, supporting the concept that *Selplg^-/-^* CD8^+^ T cells may be more sensitive to anti-PD-L1 blockade. This may be due to improved TCR signaling in Tpex cells lacking both PSGL-1 and PD-1 inhibitory signaling. We also observed that virus-specific *Selplg^-/-^* CD4^+^ T cells were significantly decreased prior to anti-PD-L1 treatment, almost undetectable in the blood of infected mice, but were reinvigorated after treatment more than WT CD4^+^ T cells. It is significant that both *Selplg^-/-^* CD4^+^ and CD8^+^ T cells were reinvigorated more than WT T cells after anti-PD-L1 treatment, indicating that targeting PSGL-1 and PD-1/PD-L1 may increase ICI efficacy by boosting both CD4^+^ T cell helper function and CTL activity. Indeed, CD4^+^ T cell help is key in curtailing chronic viral replication, therefore increasing their function is anticipated to improve antigen clearance (15). While we only targeted the PD-1/PD-L1 pathway in our studies, our findings highlight the possibility that blocking other immune checkpoints such as CTLA-4, TIM-3, and LAG-3 in combination with PSGL-1 may be a new strategy to reinvigorate exhausted T cells and increase ICI efficacy. Indeed, targeting the PSGL-1 immune checkpoint has been shown to improve anti-tumor immunity in melanoma (52).

We showed that while PSGL-1 restrained virus-specific CD4^+^ and CD8^+^ T cells during chronic viral infection, it was also necessary for their maintenance in the persistently infected host. Furthermore, *Selplg^-/-^* CD8^+^ T cells were increased in aggressive B16 melanoma tumors showing that PSGL-1 inhibited the anti-tumor T cell response. Even though virus-specific *Selplg^-/-^* CD4^+^ and CD8^+^ T cells were decreased in Cl13 mice, they were more responsive to anti-PD-L1 therapy as shown by their increased expansion and function after treatment. We found that *SELPLG* expression was increased in virus-specific CD8^+^ T cells in patients with chronic hepatitis C infection, whereas levels decreased in patients that cleared the virus, indicating that PSGL-1 is part of the T exhaustion phenotype in patients. This finding can also be explained by the difference in the status of T cell activation between cured and chronically infected patients, since *SELPLG* is increased in activated T cells. Our findings indicate that targeting PSGL-1 may hold therapeutic potential alone or in combination with other ICIs to reinvigorate exhausted T cells in chronically infected or cancer patients.

## Materials and Methods

### Mice

C57BL/6J were purchased from Jackson Laboratory and then bred in specific-pathogen-free (SPF) facilities and maintained in biosafety level 2 (BSL-2) facilities after infection in the vivarium at UC Irvine. P14 and SMARTA TCR transgenic mice were obtained from The Scripps Research Institute (originally from Dr. Charles D. Surh). These mice were bred to Ly5.1 (B6.SJL-Ptprc^a^ Pepc^b^/BoyJ) mice and to Thy1.1 (B6.PL-Thy 1^a^/CyJ). *Selplg^-/-^* mice were purchased from Jackson Laboratory and bred with the TCR transgenic mice in house. Both female and mice were used and greater than 6 weeks of age. All experiments were approved by the animal care and use committees at UC Irvine (AUP-18-148).

### Virus Infection and Titers

LCMV Clone 13 (Cl13) strain was propagated in baby-hamster kidney cells and titrated on Vero African-green-monkey kidney cells. Frozen stocks were diluted in Vero cell media and mice were infected by intravenous injection of 2x10^6^ plaque-forming units (PFUs) of LCMV Cl13.

### Adoptive Transfer

Naïve WT or *Selplg^-/-^* P14 and SMARTA T cells were isolated from mouse spleens by magnetic sorting (Stem Cell Technologies, using negative selection) in accordance with the manufacturer’s protocol. WT and *Selplg^-/-^* P14 and SMARTA T cells were transferred in equal numbers (1x10^3^ P14 and 2x10^3^ SMARTA) in 200µL PBS into naïve C57BL/6 mice by intravenous (i.v) injection. One day later, WT mice were infected with 2x10^6^ PFU LCMV Cl13 by i.v. injection. For the melanoma tumor model, naïve WT or *Selplg^-/-^* P14 T cells were isolated from mouse spleens by magnetic sorting (Stem Cell Technologies, using negative selection) according to the manufacturer’s protocol. WT and *Selplg^-/-^* P14 T cells were transferred in equal numbers (1x10^6^) in 200µL of PBS into naïve C57BL/6 mice by intravenous injection (i.v). One day later, mice were injected with 200µL of 1x10^6^ B16-GP_33_ tumor cells by subcutaneous (s.c) injection.

### Flow Cytometry

Cells from the spleens, lymph nodes (inguinal, axial, brachial), and tumors were dissociated in HBSS. For cell surface staining, 2x10^6^ cells were incubated in staining buffer (PBS, 2% fetal bovine serum (FBS) and 0.01% NaN3) for 20 minutes at 4°C with antibodies for expression of surface proteins at a 1:200 dilution. For intranuclear transcription factor staining, cells were fixed and permeabilized using a Foxp3/transcription factor fixation/permeabilization kit (Fisher) according to manufacturer’s protocol. For functional assays, cells from infected mice were cultured for 4 hours at 37°C with 2ug/mL of GP_33-41_ or GP_61-80_ peptides (AnaSpec) in the presence of brefeldin A (1µg/ml; Sigma-Aldrich), and IL2 (50u/ml). The cells were then stained with antibodies for expression of surface proteins, fixed and permeabilized using a Cytofix/Cytoperm kit (BD Bioscience), and stained with antibodies for intracellular cytokine detection at a 1:100 dilution. To evaluate cell degranulation, splenocytes were incubated with anti-CD107α. The culture media used for the 4-hour incubation was RPMI-1640 containing 10 mM HEPES, 1% non-essential amino acids and L-glutamine, 1mM sodium pyruvate, 10% heat-inactivated FBS, and penicillin/streptomycin antibiotics. The following antibodies were used in this study: Biolegend CD8 (53-6.7), CD4 (RM4-5), Vα2 (B20.1), CD90.1(OX-7), CD45.1 (A20), CD107α (1D4B), IFNγ (XMG1.2), TNFα (MP6-XT22), PD-1 (RMP1-30), TIM-3 (RMT3-23). Cell Signaling Technology TCF-1 (C63D9), Miltenyi Biotec TOX (REA473). BD Biosciences Vb8.1.2 (MR5-2), Ly-108 (13G3), Ki67 (RUO). Invitrogen GranzymeB (GB12).

### Anti PD-L1 Blockade

200μg (200µL PBS) of rat anti-mouse PD-L1 (10F.9G2) was administered by intra peritoneal (i.p) injection five separate times in WT mice. Antibody injections started at 30 days post Cl13-infection and continued every three days until 42 days post infection (30-, 33-, 36-, 39-, and 42-dpi). *In vivo* mAb were purchased from BioXcell (New Hampshire, USA). For the tumor study, WT mice were co-injected with WT and *Selplg^-/-^* P14^+^ T cells (1x10^6^ cells of each) i.v. and then injected with 1x10^6^ B16-GP_33_ melanoma cells s.c. IgG (200µg) or anti-PD-L1(200µg) (10F.9G2) was injected i.p. at day 8, 10, 12 post melanoma cell injection and mice euthanized at 15 days post melanoma cell injection.

### B16-GP_33_ Melanoma Tumor Processing

WT and *Selplg^-/-^* P14^+^ T cells were transferred in equal numbers (1x10^6^) in 200µl into naïve C57BL/6 mice by intravenous injection (i.v). One day later, mice were injected with 200µl of 1x10^6^ B16-GP_33_ tumor cells by subcutaneous injection (s.c). At day 15 after B16-GP_33_ injection, tumors were excised, minced, and digested in gentleMACS C Tubes for 40 minutes at 37°C using a gentleMACS Dissociator (Miltenyi Biotec). Tumor digestions were then passed through a 70-μm cell strainer to obtain a single-cell suspension. Cells were plated at 2x10^6^ in 96 well plates and stained for flow cytometry.

### Human HCV Data Analysis

Low-input RNA sequencing of HCV-specific CD8^+^ T cells was performed by Hensel et al., 2021 (31) [GSE150345]. Briefly, HCV-specific CD8^+^ T cells transcriptome analysis was done using an Illumina NextSeq 500 platform. For our analysis, RNA reads were aligned to the human reference genome and post-processing of the low-input sequencing data was performed at the Institute for Genomics and Bioinformatics (UCI IGB) at UC Irvine. Gene expression levels were compared in chronically infected HCV patients and HCV spontaneous resolved (SPR) HCV patients.

### Data Analysis

Flow cytometry data was analyzed with FlowJo software. Graphs were made using GraphPad Prism software. GraphPad Prism was used for statistical analysis to compare outcomes using a two-tailed unpaired *t-test*. Significance was set to p <0.05 and represented as *<0.05, **<0.001, ***<0.005, and ****<0.0001. Error bars show standard error of the mean (s.e.m.).

## Data Availability Statement

The original contributions presented in the study are included in the article/supplementary material. Further inquiries can be directed to the corresponding author.

## Ethics Statement

The animal study was reviewed and approved by UCI IACUC.

## Author Contributions

RT conceived, directed, and obtained funding for the project. KMV and RT conceptualized, designed, and analyzed the experiments and wrote the manuscript. KMV, ENN, and JMD performed the experiments. All authors provided feedback and approved the manuscript.

## Funding

This work was supported by the National Institutes of Health (R01 AI13723 to RT), Department of Defense (W81XWH-18-1-0738 to RT), The Melanoma Research Alliance (571135 to RT), and in part by (American Cancer Society Institutional Research Grant IRG-16-187-13 to RT) from the American Cancer Society, NIH IMSD training grant (GM055246 to KV), T32 Microbiology and Infectious Diseases training grant (T32AI141346 to KV), T32 virus-host interactions: a multi-scale training program (T32AI007319 to EN), and T32 Training Program for Interdisciplinary Cancer Research IDCR (T32CA009054 to JD). UCI Genomics High Throughput Facility is a Chao Family Comprehensive Cancer Center (CFCCC) shared resource supported by the Cancer Center Support Grant (P30CA062203).

## Conflict of Interest

The authors declare that the research was conducted in the absence of any commercial or financial relationships that could be construed as a potential conflict of interest.

## Publisher’s Note

All claims expressed in this article are solely those of the authors and do not necessarily represent those of their affiliated organizations, or those of the publisher, the editors and the reviewers. Any product that may be evaluated in this article, or claim that may be made by its manufacturer, is not guaranteed or endorsed by the publisher.
